# Supplementary figures and images for: Short- and long-term effects of amoxicillin/clavulanic acid or doxycycline on the gastrointestinal microbiome of growing cats
Source: PLoS One. 2021 Dec 15;16(12):e0253031. doi: 10.1371/journal.pone.0253031 (PMC8673677; doi:10.1371/journal.pone.0253031)

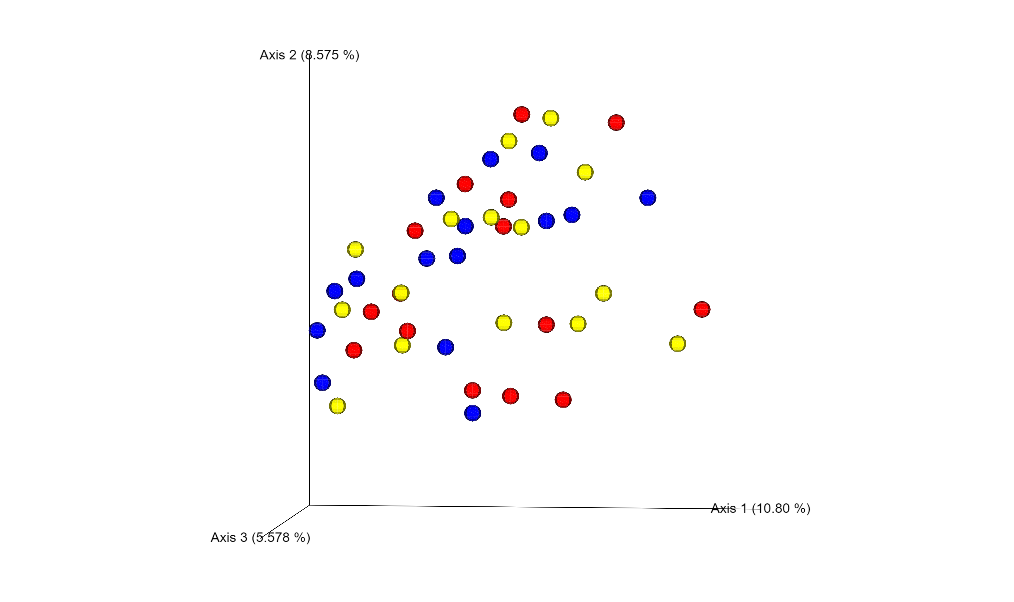

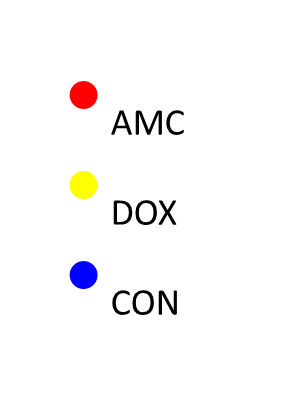

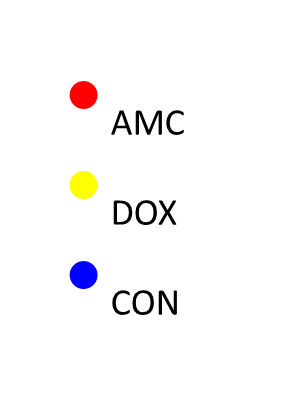

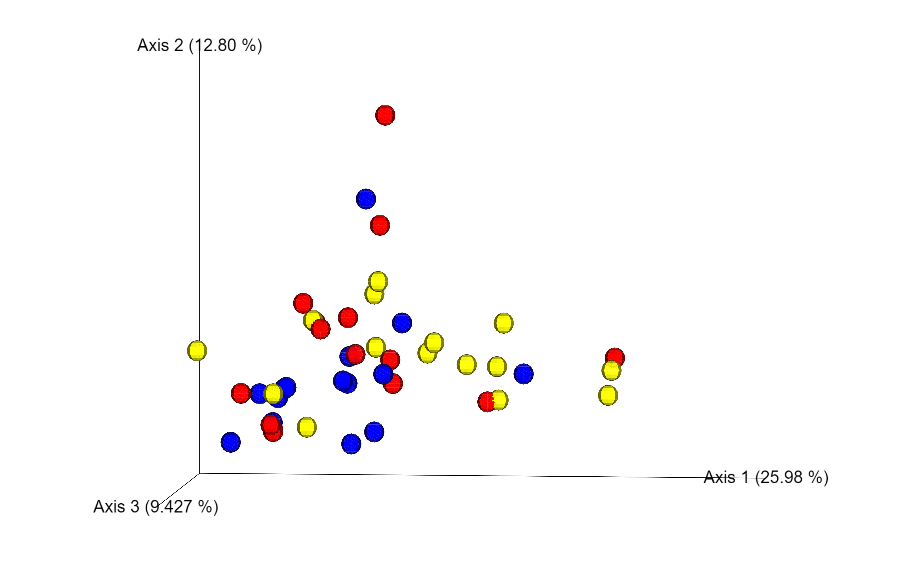

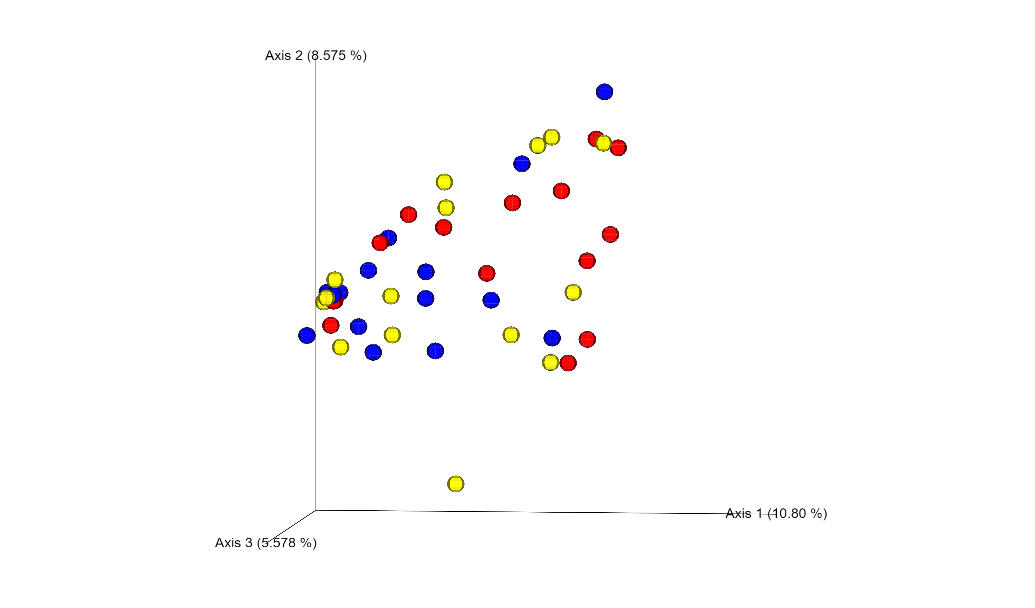

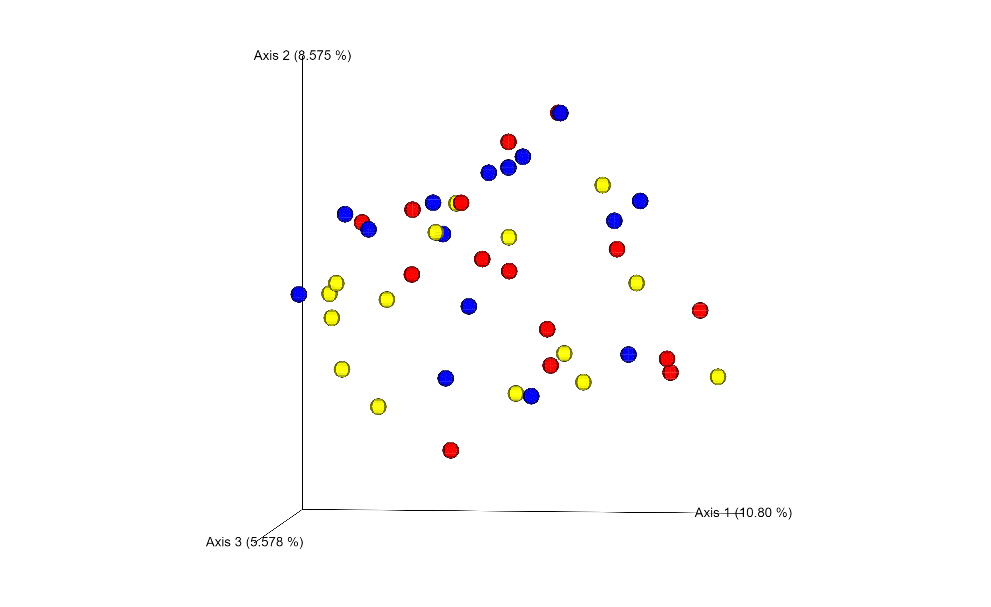

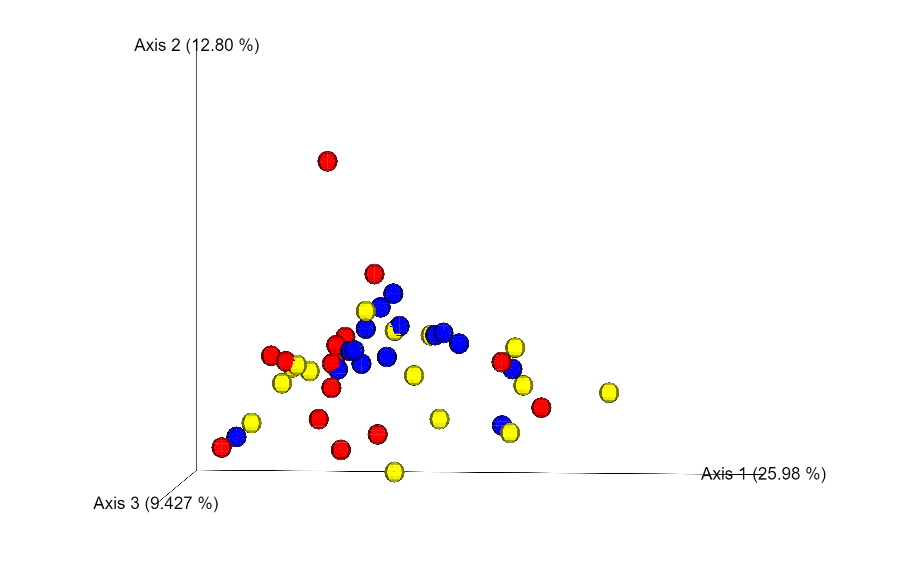

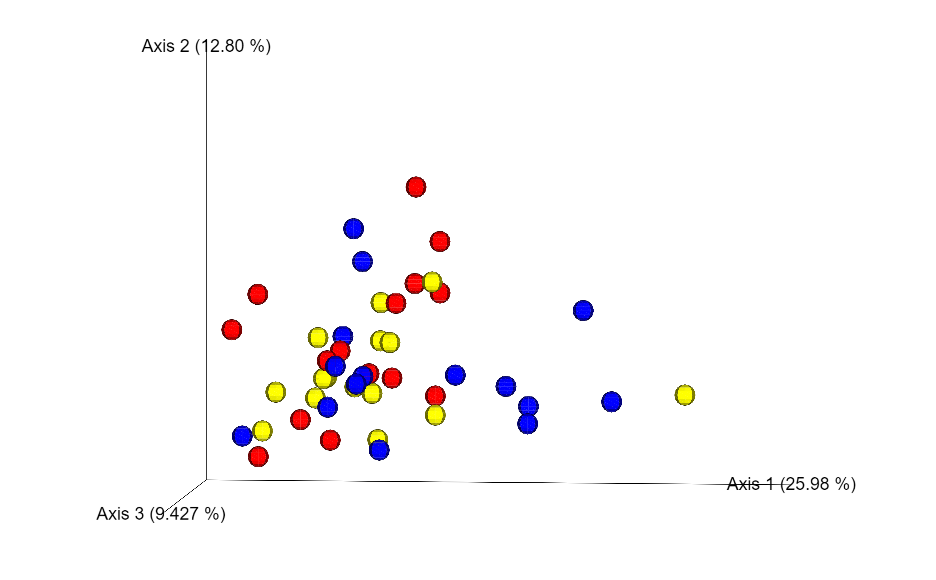

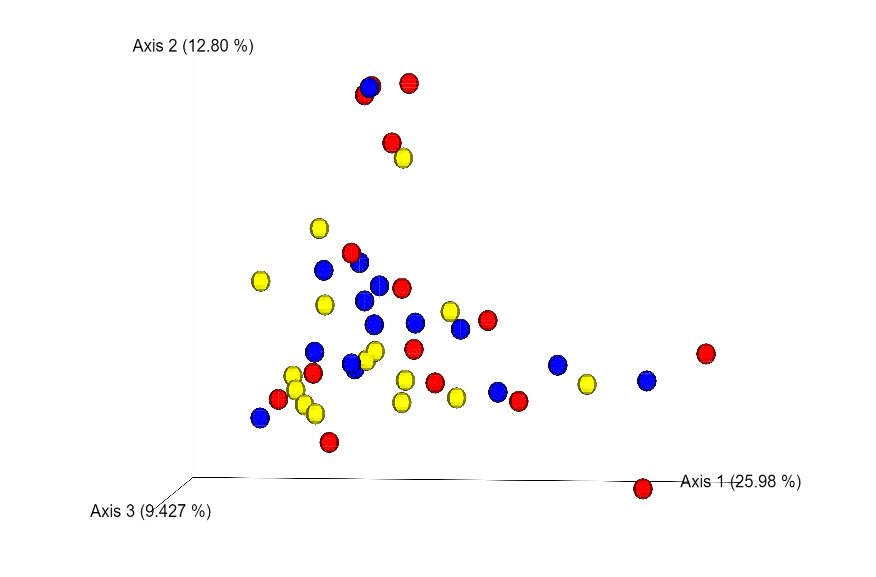

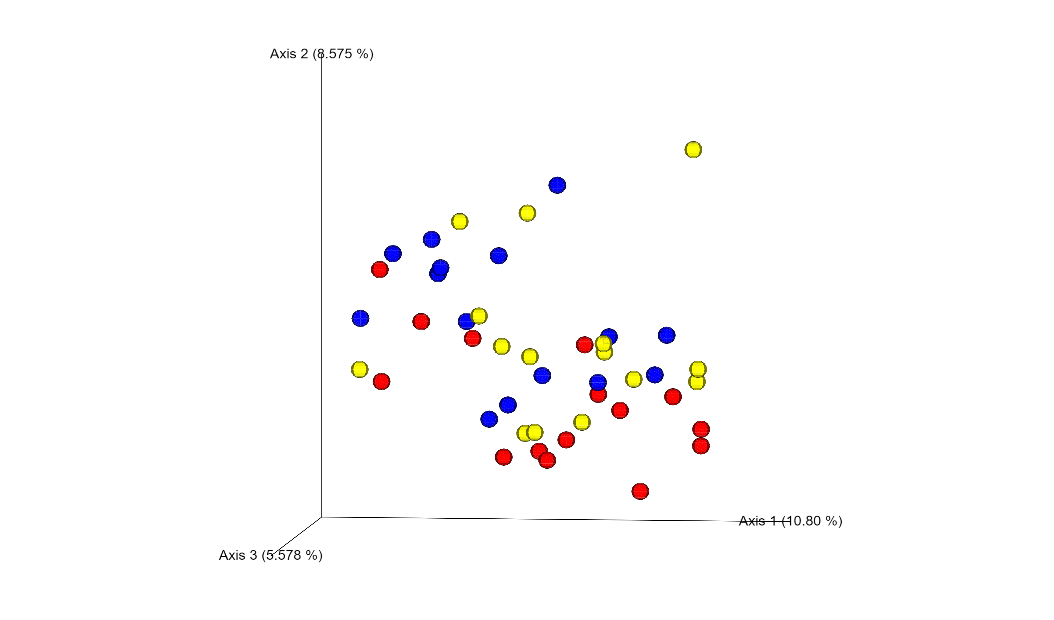


AMC

DOX

CON

Day 300

Day 60

AMC

DOX

CON

Day 20/28

Day 120

Day 120

Day 300

B

Day 60

Day 20/28

A

Supplement: S1 Fig — A) Principal Coordinate Analysis of unweighted UniFrac distances of 16S rRNA genes representing the difference in microbial communities among cats treated with amoxicillin clavulanic acid (blue circles), cats treated with doxycycline (yellow circles), and healthy control cats (red circles) on days 20/28 (last day of treatment), 60, 120, and 300. B) Principal Coordinate Analysis of weighted UniFrac distances of 16S rRNA genes representing the difference in microbial communities among cats treated with amoxicillin clavulanic acid (blue circles), cats treated with doxycycline (yellow circles), and healthy control cats (red circles) on days 20/28 (last day of treatment), 60, 120, and 300. (DOCX) [file pone.0253031.s001.docx]
