# Supplementary material for: Short- and long-term effects of amoxicillin/clavulanic acid or doxycycline on the gastrointestinal microbiome of growing cats
Source: PLoS One. 2021 Dec 15;16(12):e0253031. doi: 10.1371/journal.pone.0253031 (PMC8673677; doi:10.1371/journal.pone.0253031)
Supplement: Supplementary file 2 [file pone.0253031.s002.docx]

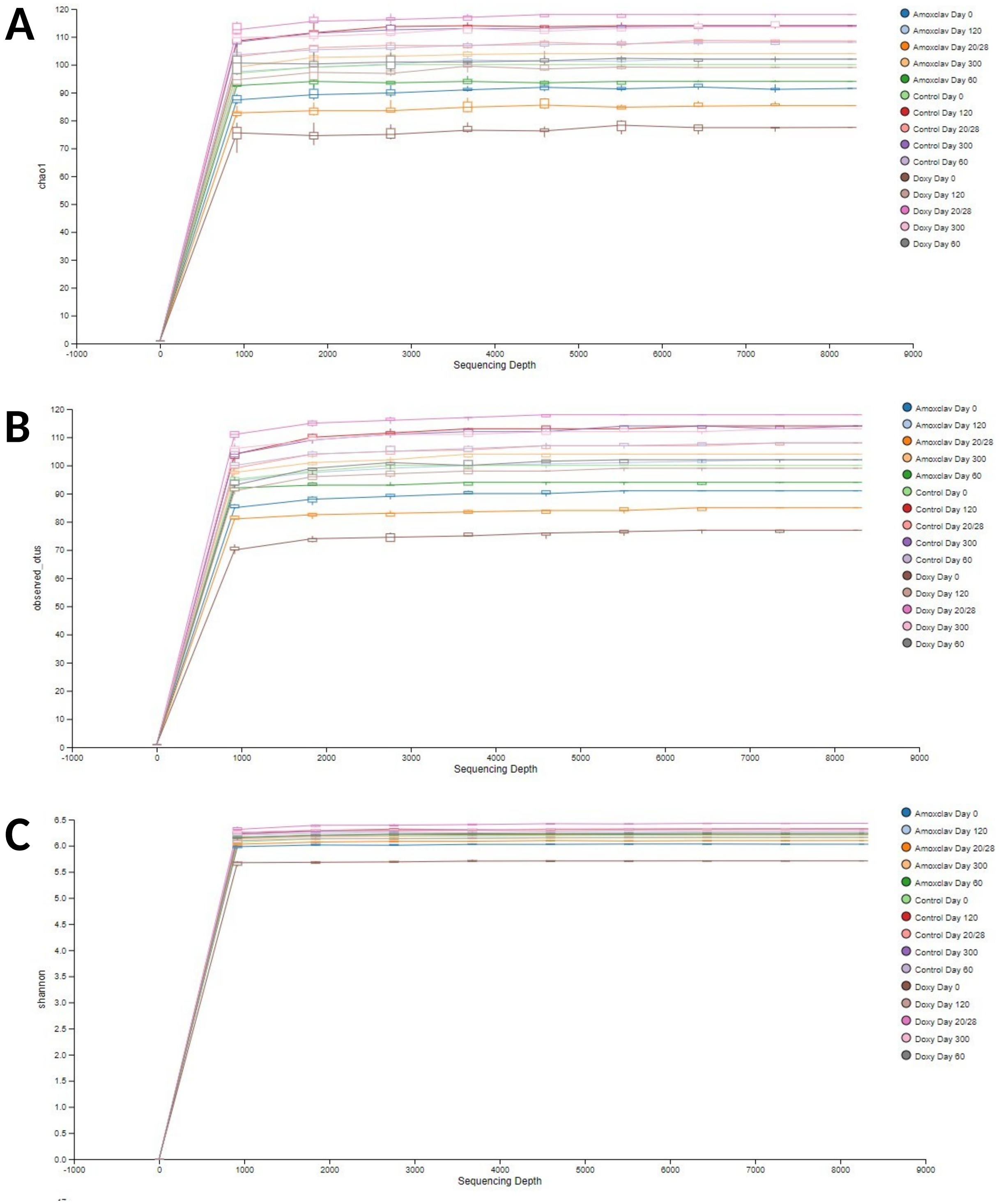
A
chao1
Sequencing Depth
-1000
0
1000
2000
3000
4000
5000
6000
7000
8000
9000
Amoxclav Day 0
Amoxclav Day 120
Amoxclav Day 20/28
Amoxclav Day 300
Amoxclav Day 60
Control Day 0
Control Day 120
Control Day 20/28
Control Day 300
Control Day 60
Doxy Day 0
Doxy Day 120
Doxy Day 20/28
Doxy Day 300
Doxy Day 60
B
observed_otus
Sequencing Depth
Amoxclav Day 0
Amoxclav Day 120
Amoxclav Day 20/28
Amoxclav Day 300
Amoxclav Day 60
Control Day 0
Control Day 120
Control Day 20/28
Control Day 300
Control Day 60
Doxy Day 0
Doxy Day 120
Doxy Day 20/28
Doxy Day 300
Doxy Day 60
C
shannon
Sequencing Depth
Amoxclav Day 0
Amoxclav Day 120
Amoxclav Day 20/28
Amoxclav Day 300
Amoxclav Day 60
Control Day 0
Control Day 120
Control Day 20/28
Control Day 300
Control Day 60
Doxy Day 0
Doxy Day 120
Doxy Day 20/28
Doxy Day 300
Doxy Day 60
